# Leptin concentration in breast milk and its relationship to duration of lactation and hormonal status

**DOI:** 10.1186/1746-4358-1-21

**Published:** 2006-11-17

**Authors:** Yesim Ozarda Ilcol, Z Banu Hizli, Tanju Ozkan

**Affiliations:** 1 Department of Biochemistry, Uludag University Medical School, Bursa, 16059, Turkey; 2 Department of Pediatric Gastroenterology, Uludag University Medical School, Bursa, 16059, Turkey

## Abstract

**Background:**

Leptin, a hormone present in breast milk, is involved in energy regulation and metabolism. The objectives of this study were to assess leptin concentrations in breast milk during the first 180 days postpartum, and to determine the relationship between the concentrations of milk leptin and circulating hormone levels in lactating women.

**Methods:**

Between April 2005 and January 2006, blood and breast milk samples were collected from 160 breastfeeding women enrolled either in the first three days (n = 37; colostrum), days 4–14 (n = 27; transitional milk), days 15–30 (n = 16; early mature milk), days 31–90 (n = 37; mature milk) or days 91–180 (n = 43; late mature milk) postpartum. Milk and serum leptin levels were measured by immunoradiometric assay. Cortisol was measured by radioimmunoassay method. Serum insulin, estradiol, prolactin and thyroxine were measured by chemiluminescent immunometric method.

**Results:**

Leptin concentrations in breast milk were highest (3.28 ± 0.41 ng/ml) in colostrum, decreased during the first 180 days of lactation, showing a significant inverse relation (r = -0.694, p < 0.001) with the days of lactation. Colostrum leptin concentrations correlated with maternal serum leptin (r = 0.425, p < 0.01), cortisol (r = 0.549, p < 0.01) and thyroxine (r = -0.530, p < 0.01). Mature milk leptin concentrations correlated with maternal serum leptin (r = 0.547, p < 0.001), insulin (r = 0.331, p < 0.05) and thyroxine (r = -0.329, p < 0.01). Serum leptin concentrations correlated with serum insulin (r = 0.648, p < 0.001), estradiol (r = 0.639, *p *< 0.001), prolactin (r = 0.530, p < 0.001) and thyroxine (r = -0.327, p < 0.05) concentrations during days 1–3 postpartum. During 15–180 postpartum days, serum leptin concentrations correlated with serum insulin (r = 0.271, p < 0.01), and thyroxine (r = -0.345, p < 0.001).

**Conclusion:**

Leptin concentrations in breast milk decrease with time during lactation and show significant relationships with other maternal hormones.

## Background

Leptin, the 167 amino acid polypeptide hormone product of the obese gene (*ob*), is secreted mainly by white adipocytes and plays an important role in the regulation of energy expenditure, food intake and metabolism [1,2]. Several recent studies have shown that human breast milk contains leptin [3-12], and its concentration in breast milk correlates with maternal circulating leptin levels [3-6,11], maternal body mass index, and adiposity [4,9,11]. Animals studies have shown that leptin can pass from mother's milk into the circulation of rat pups, leptin receptors are present in gut [13] and suckling neonates are responsive to exogenous leptin treatment [14,15]. Furthermore, it has been demonstrated that breastfed infants have higher serum leptin levels than formula fed infants [16-18] and serum leptin levels in breastfed infants are positively correlated with the leptin contents of their mothers' breast milk [6]. These findings suggest that the availability of leptin from consumed breast milk may be important for normal growth and development of breastfed infants with short and long term effects [12,19,20].

This study was designed to determine the changes in breast milk leptin levels during the 6 month period (1–180 days) after birth, by measuring whole and skim milk leptin concentrations obtained from breastfeeding women during days 1–3, 4–14, 15–30, 31–90 and 91–180 postpartum. A great deal is known about the leptin levels in human breast milk from previous studies [3-12], but there have been no systematic studies characterizing the changes in the leptin concentration in milk during this period. To the best of our knowledge, there is only one human study [11] that shows that leptin levels in milk are highest at 2–3 days after birth, decrease at 4–5 days, and remain constant for 4–6 weeks [11]. In rats, milk leptin levels fall from early to mid lactation and then increase again from mid to late lactation [21]. Breast milk is the main nutritional source of leptin for breastfed infants for the first six months. Thus, it is important to know whether its concentration in breast milk varies at different stages of lactation during this period. Second, we sought to determine whether the concentrations of leptin in breast milk are related to serum leptin and other serum hormones in lactating women. It is well known that mammary differentiation and milk secretion are controlled by reproductive and metabolic hormones [22], and these hormones also affect the synthesis and secretion of leptin from white adipose tissue [23-37].

## Methods

### Subjects and study design

Between April 2005 and January 2006, breast milk and venous blood samples were obtained from healthy lactating women who gave written, informed consent to participate in the study. The study protocol, the contents of the written information sheet, and the consent form were approved by the Ethics Committee of Uludag University Medical School, Bursa, Turkey.

The study design was longitudinal and cross sectional. Twenty-two breastfeeding women [age (years) = 29 ± 2 (mean ± standard error of the mean); weight (kg) = 69 ± 3; height (cm) = 161 ± 2 cm; BMI (body mass index, kg/m^2^) = 26 ± 2] participated in the longitudinal part of the study. In the longitudinal component of the study, changes of milk leptin concentration were investigated only during the first 30 days of the lactation period due to recruitment and retainment problems of subjects for a longer period. Breast milk samples were obtained from the same women at 1–3 days, 4–14 days and 15–30 days after birth.

The cross-sectional component of the study was designed to investigate changes in milk leptin concentrations during a longer period, from 0 to 180 lactation days. Five groups of exclusively breastfeeding women participated in the cross-sectional study. The first group consisted of 37 breastfeeding women [age (years) = 30 ± 2; weight (kg) = 70 ± 3; height (cm) 160 ± 2 cm; BMI = 27 ± 2] enrolled at days 1 to 3 after birth (expressing colostrum). The second group consisted of 27 breastfeeding women [age (years) = 31 ± 2; weight (kg) = 67 ± 2; height (cm) 159 ± 2 cm; BMI = 26 ± 1] enrolled at days 4–14 after birth (expressing transitional milk). The third group consisted of 16 breastfeeding women [age (years) = 29 ± 1; weight (kg) = 69 ± 3; height (cm) 159 ± 2 cm; BMI = 27 ± 1] enrolled at days 15–30 after birth (expressing early mature milk). The fourth group consisted of 37 breastfeeding women [age (years) = 29 ± 2; weight (kg) = 66 ± 3; height (cm) 161 ± 2 cm; BMI = 25 ± 1] enrolled at days 31–90 after birth (expressing mature milk). The fifth group consisted of 43 breastfeeding women [age (years) = 30 ± 2; weight (kg) = 65 ± 3; height (cm) 159 ± 2 cm; BMI = 26 ± 1] enrolled at days 91–180 after birth (expressing late mature milk). These 5 cross-sectional maternal groups were demographically and biologically equivalent with regard to age [F(4,155) = 0.140; P = 0.967], weight [F(4,155) = 0.517; P = 0.723], height [F(4,155) = 0.193, P = 0.942] and BMI [F(4,155) = 0.364, P = 0.834]. The power of performed test with alpha equal to 0.05 was 0.049 in all of these analyses.

Inclusion criteria were women who gave birth at term after uncomplicated pregnancies. Exclusion criteria were lactation failure, any existing disease (i.e. maternal disease due to pregnancy or unrelated and pre-existing or neonatal disease), under hormonal and any drug therapy, and body mass index greater than 35.0 at enrolment.

### Milk and blood samples collection

Fore milk sample collection was standardized by milk volume and time: 5 ml were collected in plastic tubes by hand expression 3 hours (h) after breakfast (bread, butter, olive, cheese and tea). Samples were vortexed, divided into 500 μl aliquots (1.0 ml Eppendorf tubes) and frozen at -20°C until analysis. Maternal blood (5 ml) was collected into a plain evacuated glass tube at the same time as the milk sample. Blood samples were centrifuged within 30 min at 2000 × g for 10 min at 4°C. Serum was divided into 500 μl aliquots and stored at -20°C until analysis. Milk samples were thawed overnight in the refrigerator and vortexed continuously to ensure sample uniformity. Given evidence that leptin levels are higher in whole milk than in skim milk [4,5,11] and that sonication yields significantly increased levels [5,11], we analyzed whole milk samples after sonication (Bandelin Electronic, UW 70, D-1000 Berlin 45, Germany) with 8–10 bursts of 10 sec in duration each.

### Assays

We used a human leptin immunoradiometric assay kit (Human Leptin IRMA, DSL-23100; Webster, Texas, USA) for the measurements of leptin in breast milk and serum. All milk and serum samples from the individual subjects were assayed in duplicate in the same test battery. Intra- and inter- assay coefficients of variation (CV) for the leptin IRMA were < 5% and < 6.7 %, respectively, with a detection limit 0.1 ng/ml using 100 μl samples.

Chemiluminescent microparticle immunoassay (CMIA) was used for the quantitative determination of estradiol, prolactin, and thyroxine by using Architect 8200^® ^autoanalyser (Architect 8200, Abbott). Serum cortisol was measured by radioimmunoassay using a commercially available kit (Diagnostic Products Corporation, Los Angeles, CA, USA). Insulin was measured by chemiluminescent immunometric assay with an Immulite 2000^® ^Analyzer (Diagnostic Products Corporation, Los Angeles, CA, USA).

### Analysis

Statistical analysis was performed using SigmaStat V2.03 (SPSS Science Software GmbH, Erkrath, Germany) for Windows. The Kolmogorov-Smirnov test was used for normality. Differences in the log values of whole milk leptin concentrations and their changes with the lactation periods were examined with one-way ANOVA followed by Tukey's pairwise multiple comparison method. In the longitudinal study, changes in the log values of serum leptin concentrations in lactating women at various time points during 1 to 30 days lactation period were conducted with one-way repeated measure ANOVA followed by Tukey's multiple comparison method. Longitudinal changes in serum hormone levels during 180 days of lactation were examined with one-way ANOVA followed by Tukey's pairwise multiple comparison method. The relationships between two variables were determined by Pearson's correlation and multiple regression analysis. Data are given as mean ± standard error of the mean (SEM); p values less than 0.05 were considered significant in all tests.

## Results

### Leptin concentrations in breast milk during lactation

Leptin concentration in breast milk showed a lognormal distribution. There was a wide range of leptin concentrations in colostrum (0.16–7.0 ng/ml) and mature milk (0.11–4.97 ng/ml).

**Figure 1:**
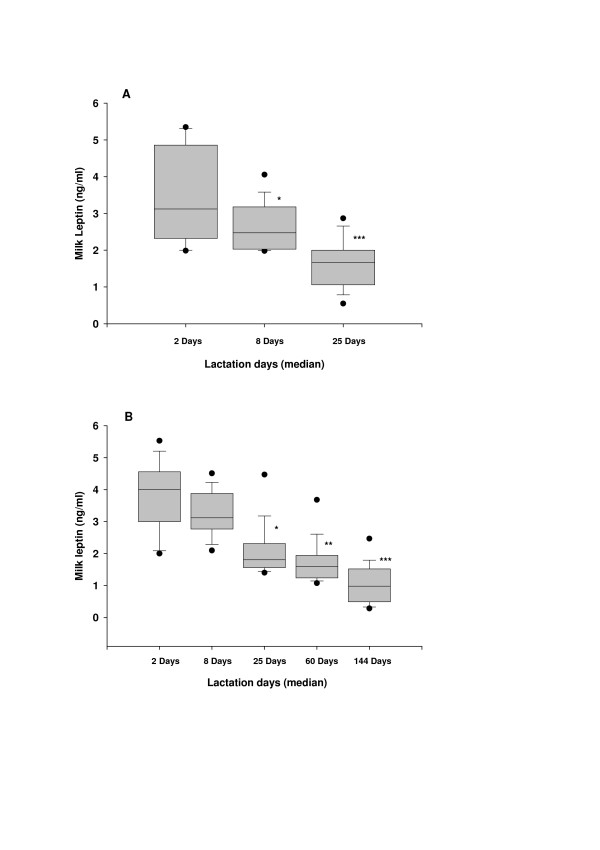
**Changes in leptin concentrations in human breastmilk during 180 days of lactation period**. **(A) **Box plot ofleptin concentrations in milk samples were obtained frombreastfeeding women taking part in the longitudinal study at 1–3 days(median = 2 days), 4–14 days (median = 8 days) at 15–30 days (median = 25 days) after birth. **(B) **Box plot of leptin concentrations in milk samples collected from 160 breastfeeding women taking part in the cross-sectional study at 1–180 days and they grouped into five intervals: 1–3 days (median = 2 days), 4–14 days (median = 8 days), 15–30 days (median = 25 days), 31–90 days (median = 60 days) and 91–180 days (median = 144 days). The filled circle at the upper and the lower part of each box show the 5^th ^and 95^th ^percentiles, respectively. *p < 0.05; **p < 0.01; ***p < 0.001 when compared with the values observed for 1–3 days.

In the longitudinal study, leptin levels in colostrum (collected at 1–3 days) were 3.35 ± 0.25 ng/ml and then fell significantly [F(2,42) = 30.058, p < 0.001; power of the performed test with alpha = 0.050: 1.000] to 2.65 ± 0.21 ng/ml (*p *< 0.05) in transitional milk (expressed at 4–14 days) and to 1.63 ± 0.18 ng/ml (p < 0.001) in early mature milk (Figure 1A).

In the cross sectional study, the profile of the changes in breast milk leptin concentrations with time was similar to the profile observed in the longitudinal study. Breast milk leptin concentrations were highest in colostrum (3.28 ± 0.21 ng/ml), breast milk expressed 1 to 3 days after birth, and then fell significantly [F(4,155) = 14.34, p < 0.001; power of the performed test with alpha = 0.050: 1.000] during the 180 days lactation period (Figure 1B). Leptin concentrations in breast milk expressed 31–90 days or 91–180 days were lower than the observed concentrations in colostrum (p < 0.001) and in transitional milk (p < 0.01). Regression analysis revealed a significant inverse relation (r = -0.694, p < 0.001; power of the performed test with alpha = 0.050: 1.000) between breast milk leptin concentrations and lactation days.

### Serum leptin, cortisol, insulin, estradiol, prolactin, and thyroxine concentrations during lactation

The mean concentrations of serum leptin, cortisol, insulin, thyroxine, estradiol, and prolactin in the five study groups are shown in Table 1. Serum leptin concentrations were highest during days 1–3, and then fell slightly [F(4,155) = 3.67, p < 0.05; power of the performed test with alpha = 0.050: 1.000] during the 180 days lactation period (Table 1).

**Table 1:** Serum leptin, cortisol, insulin, thyroxine, estradiol and prolactin concentrations during days 1–180 postpartum

Lactation period	N	Leptin	Cortisol	Insulin	Thyroxine	Estradiol	Prolactin
		(ng/ml)	(μg/dl)	(μIU/ml)	(μg/dl)	(pg/ml)	(ng/ml)
1–3 postpartum days	37	16.6 ± 1.7	38.9 ± 2.9	13.0 ± 2.3	11.9 ± 0.5	148 ± 20	264 ± 19
4–14 postpartum days	27	14.2 ± 1.9	19.2 ± 1.4*	11.6 ± 1.5	10.8 ± 0.3	47 ± 5*	155 ± 11*
15–30 postpartum days	16	13.8 ± 1.6	21.7 ± 2.2*	11.1 ± 1.0	7.9 ± 0.4*	55 ± 4*	135 ± 17*
31–90 post partum days	37	13.2 ± 1.6	17.1 ± 1.9*	15.2 ± 2.2	7.3 ± 0.3*	51 ± 8*	85 ± 9*
91–180 postpartum days	43	10.2 ± 1.4*	18.3 ± 1.1*	11.2 ± 1.3	7.3 ± 0.2*	49 ± 7*	44 ± 5*

Blood samples collected from 160 healthy lactating women at 1–180 days, grouped into five intervals: 1–3 days (colostrum), 4–14 days (transitional milk), 15–30 days (early mature milk), 31–90 days (mature milk) and 91–180 days (late mature milk).

Statistical analyses of the log values of serum leptin, cortisol, insulin, thyroxine, estradiol and prolactin concentrations were performed with one-way ANOVA followed by Tukey's multiple comparison method.

*p < 0.05-0.001 when compared with the observed values for days 1–3.

Lactating women expressing transitional (postpartum days 4–14) and mature milk (postpartum days 15 to 180) had significantly lower (p < 0.05–0.001) serum cortisol, estradiol and prolactin concentrations than lactating women expressing colostrum at postpartum days 1 to 3 (Table 1).

### Correlations between breast milk leptin and maternal serum hormone concentrations

**Figure 2:**
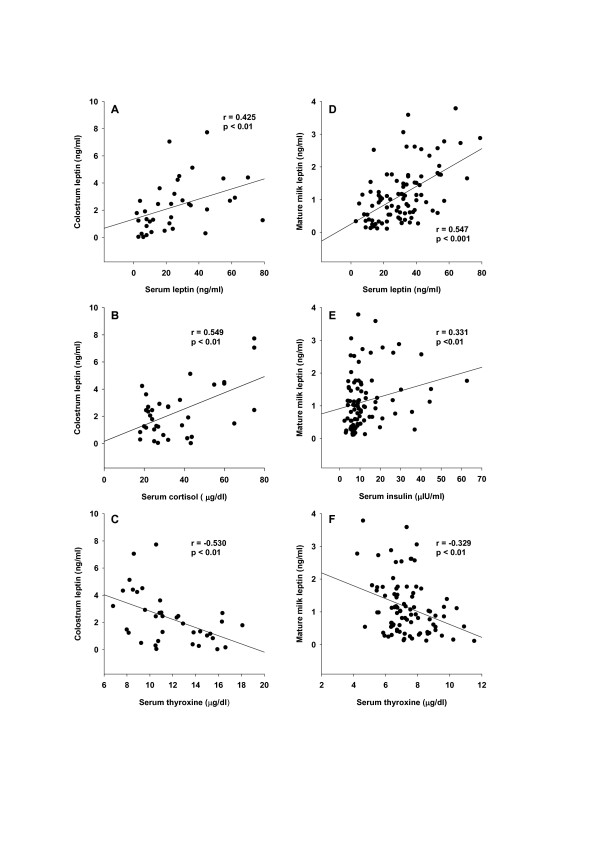
**Relationships among leptin concentrations incolostrum and mature milk and maternal serum leptin, cortisol, insulin and thyroxine concentrations**. In the cross-sectional component of the study, leptin concentrations in breast milk samples collected at 1–3 days [colostrum (Figure 2 A, B and C)] or at 15–180 days [mature milk (Figure 2 D, E and F)] were plotted against the maternal serum concentrations of leptin (A and D), cortisol (B), insulin (E) and thyroxine (C and F).

The relationships between colostrum (expressed at 0–3 days) and mature milk (expressed at 15–180 days) leptin concentrations and maternal serum hormones were determined in the cross-sectional component of the study. Regression analysis revealed significant correlations between colostrum leptin concentrations and maternal serum leptin (r = 0.425, p < 0.01), cortisol (r = 0.549, p < 0.01) and thyroxine (r = -0.0530, p < 0.01) concentrations (Figure 2A, B, C). There was no correlation between colostrum leptin concentrations and serum insulin (r = 0.159, p = 0.57), prolactin (r = 0.120, p = 0.66) or estradiol (r = -0.180, p = 0.29) concentrations (data not shown). Correlation analysis between log values of colostrum leptin concentrations and maternal serum hormones revealed similar results (data not shown).

Regression analysis revealed significant correlations between breast milk leptin concentrations during days 15–180 and maternal serum leptin (r = 0.547, p < 0.001), thyroxine (r = -0.329, p < 0.01) and insulin (r = 0.331, p < 0.01) concentrations (Figure 2D, E, F). There was no correlation between mature milk leptin concentrations and maternal serum cortisol (r = 0.119, p = 0.25) prolactin (r = 0.130, p = 0.24) or estradiol (r = 0.061, p = 0.56) concentrations (data not shown). Correlation analysis between log values of mature milk leptin concentrations and maternal serum hormones revealed similar results (data not shown).

**Figure 3:**
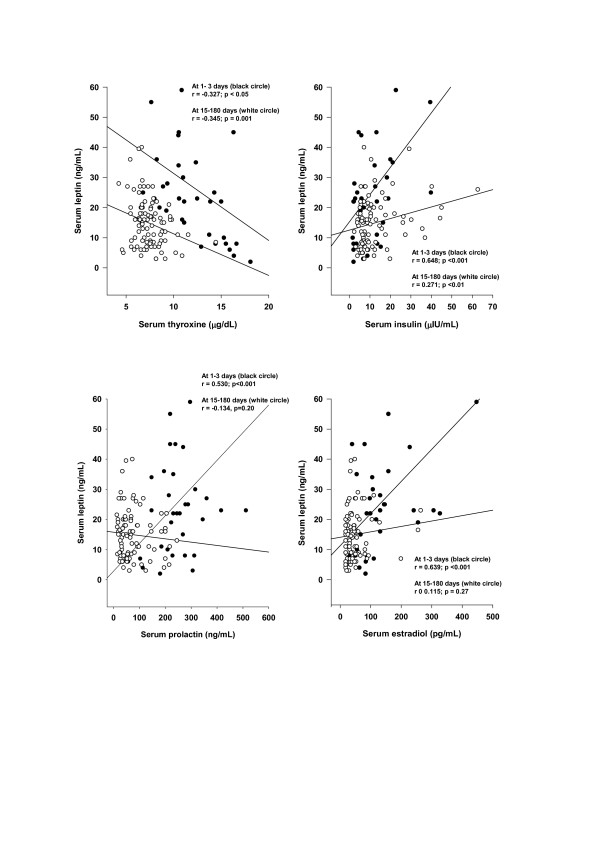
**Relationships among serum leptin concentrations and serum thyroxine, insulin, prolactin and estradiol concentrations in lactating women during days 1–3 or 15–180 postpartum**. Serum leptin concentrations were plotted against serum thyroxine, insulin, prolactin and estradiol concentrations in venous blood samples collected from breastfeeding women at days 1–3 (white circle) and 15–180 (black circle) postpartum.

Serum leptin concentrations were correlated with serum thyroxine (r = -0.327, p < 0.05), insulin (r = 0.648, p < 0.001), estradiol (r = 0.639, p < 0.001) and prolactin (r = 0.530, p < 0.001) during days 1–3 postpartum (Figure 3). In the group expressing mature milk, serum leptin concentrations were also correlated with serum thyroxine (r = -0.345, p < 0.001) and insulin (r = 0.271, p < 0.01), but not with serum estradiol (r = 0.115, p = 0.27) or prolactin (r = -0.134, p = 0.20). There was no correlation between serum leptin and cortisol concentrations in breastfeeding women either expressing colostrum or mature milk (data not shown).

## Discussion

These data show that breast milk leptin concentrations decrease during the first180 days of lactation. Leptin levels in colostrum are correlated positively with the maternal serum leptin and cortisol, and are inversely correlated with serum thyroxine. Leptin levels in mature breast milk are correlated with maternal serum leptin, insulin and thyroxine, but not with serum cortisol, estradiol and prolactin. Serum leptin concentrations in lactating women during days 1–3 and 15–180 postpartum were inversely correlated with serum thyroxine. At 1–3 days, but not at 15–180 days serum leptin concentrations were also positively correlated with serum prolactin and estradiol.

Our observation that higher leptin levels are found in colostrum than in mature milk agrees with a recent report [11] showing that leptin levels in colostrum (at 2–3 days after birth) are significantly higher than in transitional (at 4–5 days after birth) or mature (4–6 weeks after birth) milk. The data from recent study on human breast milk leptin concentrations show that the breast milk leptin concentrations at the third month are about 30 to 50% of the milk leptin concentrations observed at the 15^th ^day in the same lactating women [12]. In the present study we observed that breast milk leptin concentrations are highest at 1–3 days and lowest at 91–180 days during the first180 days of lactation (Figure 1). We also observed that there is a negative correlation (r = -0.694; p < 0.001) between whole breast milk leptin levels and the days of lactation. Taken together, these data indicate that leptin content of breast milk decreases with time. It is known, however, that milk transfer to the infant is low on days 1 and 2 and increases gradually during next 180 days [38,39]. Thus it is reasonable to assume that the amount of leptin delivered to the infant by breast milk may be compensated, at least partly, by increasing the breast milk volume per day.

The leptin levels observed in the current study in breast milk were comparable to most reported values [3,4,11], but much lower than the values mentioned in a recent report [12]. As noted previously [3,11], we observed that the content of leptin in colostrum and in mature breast milk varies considerable among lactating, breastfeeding mothers. It has been demonstrated [6] that serum leptin levels in breastfed infants are positively correlated with the leptin contents of their mothers' breast milk. It has also been shown that weight gain of newborns between days 15 and 30 correlated with breast milk leptin levels on the 15^th ^day [12]. Data from animal studies show that exogenously administering leptin to nursing mothers leads to a rise in the breast milk leptin levels, and an increase in the leptin detected in the gastrointestinal systems and blood streams of suckling pups [3]. Furthermore suckling neonates are responsive to exogenous leptin treatment [14,15]. Together, these data suggest that the consumption of breast milk with different leptin content can directly affect circulating leptin levels, and may regulate neonatal metabolism, in breastfed infants.

We found here that leptin concentrations, in both colostrum and mature human milk, were inversely related with maternal serum thyroxine levels. Maternal serum thyroxine levels at days 1–3 postpartum were higher (Table 1), and the relationship between circulating and colostrum leptin concentrations was stronger than that seen with mature milk (Figure 2). Furthermore, serum leptin concentrations in breastfeeding mothers were inversely related to the maternal serum thyroxine levels (Figure 3). Taken together, these data suggest that thyroxine might have physiological inhibitory effects on milk and serum leptin levels in lactating women. A negative influence of thyroid hormones on serum leptin concentrations has also been observed in experimental rat studies [40-43]. Although many studies in humans on serum leptin levels during thyroid dysfunction revealed conflicting results [44-50], a recent report [50], also supports an inverse relationship between thyroid activity and circulating leptin concentrations.

Although numerous studies have shown that insulin [23-29], glucocorticoids [23,25,30-32] and prolactin [33,34] act on the adipose tissue to increase leptin synthesis and secretion, the influence of these hormones on serum and milk leptin levels has been determined only in few studies involving relatively small numbers of lactating women. In a study of 18 lactating women during days 3 to 120 after birth, no relationship was found between milk leptin concentrations and maternal serum insulin levels [6]. Another study performed in 23 lactating women also showed no relation between milk leptin and serum insulin levels [4]. Butte et al. found a positive correlation between serum leptin and insulin, and a negative correlation between serum leptin and prolactin concentrations in 39 lactating women at 3 and 6 months postpartum [37]. In the present study, leptin concentrations in colostrum were correlated with the maternal serum cortisol levels, but not serum insulin, estradiol or prolactin levels (Figure 2). On the other hand, leptin concentrations in mature milk were correlated positively with maternal serum insulin levels, but not with serum cortisol, estradiol or prolactin levels (Figure 2). Serum leptin concentrations in lactating women in the first three days postpartum, and during days 15–180 postpartum were positively correlated with serum insulin levels. During the first three days, serum leptin concentrations were also positively correlated with serum prolactin levels (Figure 3). The observed positive correlation between serum leptin and serum estradiol in lactating women during days 1 to 3 after birth also shows that estradiol may increase serum leptin levels at the early stage of the lactation, as expected [35,36].

## Conclusion

This study shows that breast milk leptin content varies considerably during lactation. Furthermore, this study also provides the first correlations between leptin concentrations in breast milk and serum levels of leptin, insulin, thyroxine, cortisol, estradiol and prolactin. The relationships between the concentrations of milk leptin and maternal serum hormones vary during lactation.
